# Naproxen in the environment: its occurrence, toxicity to nontarget organisms and biodegradation

**DOI:** 10.1007/s00253-019-10343-x

**Published:** 2020-01-10

**Authors:** Danuta Wojcieszyńska, Urszula Guzik

**Affiliations:** Institute of Biology, Biotechnology and Environmental Protection, Faculty of Natural Science, University of Silesia in Katowice, Jagiellońska 28, 40-032 Katowice, Poland

**Keywords:** Naproxen, Microorganisms, Toxicity, Biodegradation

## Abstract

**Abstract:**

This article summarizes the current knowledge about the presence of naproxen in the environment, its toxicity to nontarget organisms and the microbial degradation of this drug.

Currently, naproxen has been detected in all types of water, including drinking water and groundwater. The concentrations that have been observed ranged from ng/L to μg/L. These concentrations, although low, may have a negative effect of long-term exposure on nontarget organisms, especially when naproxen is mixed with other drugs. The biological decomposition of naproxen is performed by fungi, algae and bacteria, but the only well-described pathway for its complete degradation is the degradation of naproxen by *Bacillus thuringiensis* B1(2015b). The key intermediates that appear during the degradation of naproxen by this strain are O-desmethylnaproxen and salicylate. This latter is then cleaved by 1,2-salicylate dioxygenase or is hydroxylated to gentisate or catechol. These intermediates can be cleaved by the appropriate dioxygenases, and the resulting products are incorporated into the central metabolism.

**Key points:**

•*High consumption of naproxen is reflected in its presence in the environment.*

*•Prolonged exposure of nontargeted organisms to naproxen can cause adverse effects.*

*•Naproxen biodegradation occurs mainly through desmethylnaproxen as a key intermediate.*

## Introduction

Naproxen, which is a bicyclic propionic acid derivative, is a widely known drug from the group of non-selective, non-steroidal anti-inflammatory drugs (Dzionek et al. 2018). Its mechanism of action is based on the inhibition of both cyclooxygenase isoforms that are involved in the synthesis of prostaglandins, prostacyclin and thromboxane from arachidonic acid (Angiolillo and Weisman 2017; Barcella et al. 2019). The advantages of naproxen are its rapid absorption and its long duration of action, which result from its long biological half-life (approximately 13 h), and its ability to strongly bind to the plasma proteins. Another advantage is also its diffusion into the synovial fluid. It is the preferred drug for treating osteoarthritis in patients who are at a high cardiovascular risk because, unlike diclofenac and other NSAIDs including the selective ones, when it is used in high doses, it poses a lower vascular risk (Angiolillo and Weisman 2017; Barcella et al. 2019). These advantages together with fact that naproxen can be purchased without a prescription translate into its popularity on the pharmaceutical market. For more than 40 years, it has held a strong position compared to other NSAIDs (Aguilar et al. 2019; Dzionek et al. 2018). In 2003, almost 3000 t of naproxen were produced in the world (Li et al. 2016). According to ClinCalc  (2019), it was prescribed 11,470,076 times in the USA in 2016. The popularity of naproxen in the treatment of pain has resulted in its occurrence in the environment (Aguilar et al. 2019; Madikizela et al. 2017; Xu et al. 2019).

## Occurrence of naproxen in the environment

**Fig. 1:**
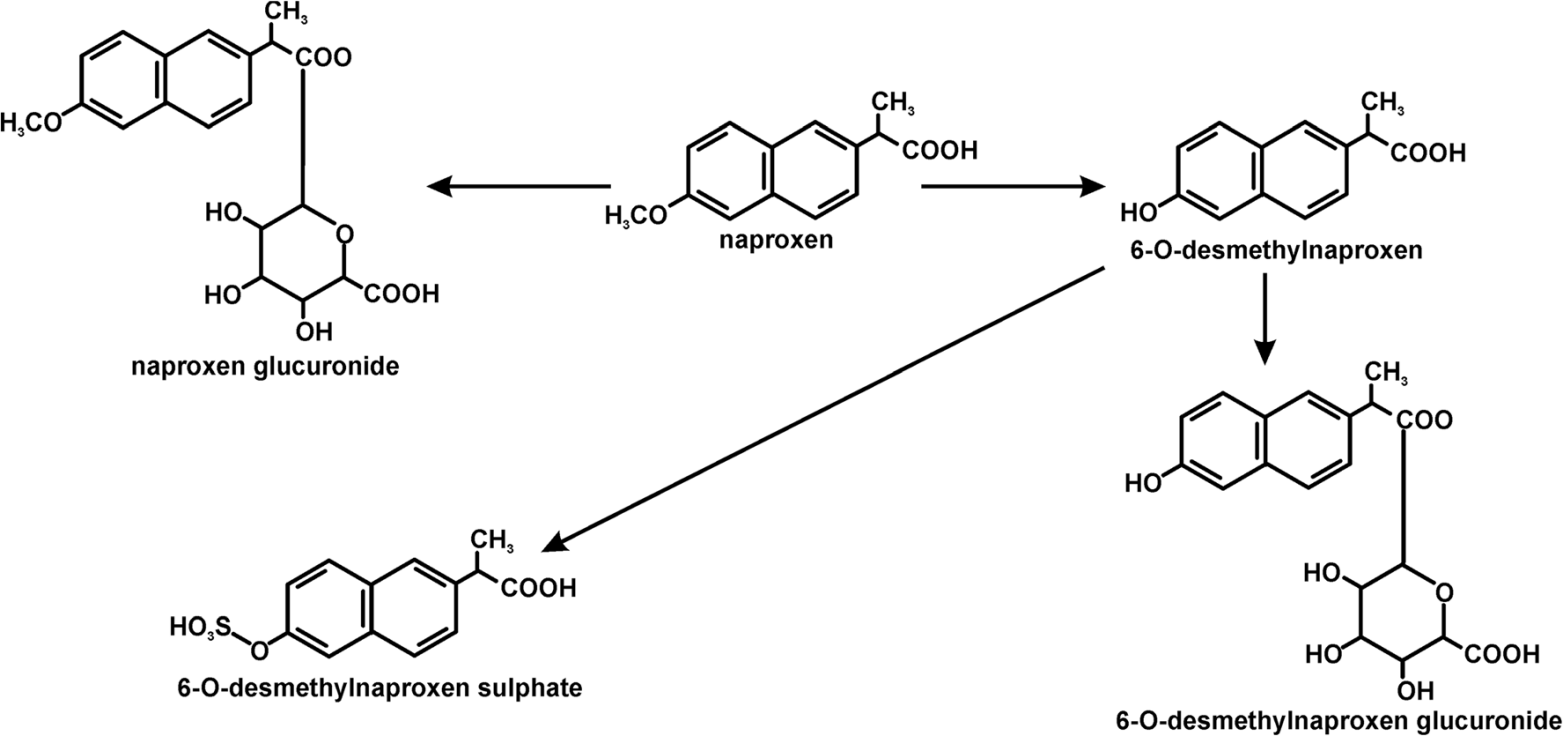
The transformations of naproxen that occur in higher organisms (Addison et al. 2000)

Since naproxen entered the market in 1976, it has enjoyed unflagging popularity. This has resulted in the occurrence of this compound in wastewater (Garcia-Medina et al. 2015; Shanmugam et al. 2014). In the body, naproxen is metabolized into two key products: O-desmethylnaproxen and naproxen glucuronide (Fig. 1) (Addison et al. 2000). Therefore, these compounds also end up in the wastewater. Research to date has indicated that in sewage treatment plants, naproxen does not undergo complete mineralization and despite a relatively high degree of transformation goes into the environment along with the outflow from a treatment plant (Grenni et al. 2014; Lahti and Oikari 2011; Marco-Urrea et al. 2010). The removal of naproxen in wastewater treatment plants is significantly different and ranges from its almost complete removal to only a 40% degradation level (Marco-Urrea et al. 2010). It was observed that in the effluents of wastewater treatment plants, the concentration levels of naproxen ranged from 25 ng/l to 33.9 μg/l (Marotta et al. 2013). Moreover, Suzuki et al. (2014) showed that the effluents of wastewater treatment plants also contain its major metabolite – 6-O-desmethylnaproxen at a concentration of 0.56 μg/l. Because of incomplete decomposition, naproxen occurs in groundwater, surface water as well as in drinking water (Benotti et al. 2009). It was found in 69% of >100 water samples from more than 100 European rivers at a concentration of up to 2.027 μg/l (Ding et al. 2017). Recent investigations of European Union waters have indicated that concentration of naproxen in wastewater treatment plants and in surface waters exceeds the concentration that is recommended by the European Medicines Agency by 10- to 500-fold (Grenni et al. 2013). The naproxen concentrations that have been detected in the environment are presented in Table 1.

**Table 1:** Naproxen concentration in the aquatic environment

Sources	Concentration (μg/l)	References
Estuary of Seine River, France	Up to 0.275	Shanmugam et al. 2014
German rivers	Up to 0.39	Vulava et al. 2016
Lake Greifensee, Switzerland	0.003–0.010	Straub and Stewart 2007
Lake Haapajarvi, Finland	0.040–0.21	Brozinski et al. 2013
A major river in Korea	0.326	Ji et al. 2013
Marina catchment, Singapore	0.008–0.108	Shanmugam et al. 2014
Ebro River, Spain	Up to 0.109	Shanmugam et al. 2014
Elbe River, Germany	up to 0.032	Straub and Stewart 2007
Fyris River, Sweden	0.447	Shanmugam et al. 2014
Glatt River, Switzerland	0.004–0.36	Straub and Stewart 2007
Han River, South Korea	0.005–0.1	Shanmugam et al. 2014
Ladysmith River, South Africa	2.77	Madikizela et al. 2017
Lopan River, Ukraine	0.2–0.264	Shanmugam et al. 2014
Malir River, Pakistan	11.4–32.00	Shanmugam et al. 2014
Mbokodweni River, South Africa	1.0–3.8	Sibeko et al. 2019
Pearl River, China	Up to 0.328	Shanmugam et al. 2014
Rhine River, Germany and Switzerland	Up to 0.028	Straub and Stewart 2007
Sindian River, Taiwan	0.035–0.27	Shanmugam et al. 2014
Tiber River, Italy	0.24–0.27	Grenni et al. 2014
Rivers, Canada	Up to 4.5	Shanmugam et al. 2014
Rivers, Japan	Up to 0.24	Shanmugam et al. 2014
Rivers, Poland	Up to 0.753	Shanmugam et al. 2014
Rivers, Slovenia	Up to 0.08	Shanmugam et al. 2014
Seawater, Portugal	0.178	Jallouli et al. 2016
Sediment of the Danube River, Europe	7–57 μg/kg	Garcia-Medina et al. 2015
Water sources from Mexico City, Mexico	0.052–0.186	Garcia-Medina et al. 2015

The concentration of naproxen in the environment depends on its physicochemical properties such as solubility and chemical stability as well as environmental properties, while its mobility in the environment correlates to chemical properties such as the dissociation constant and the value of the octanol-water partition coefficient (log*K*_*ow*_) (Kim and Zoh 2016; Sibeko et al. 2019). The value of this coefficient for naproxen (3.2) indicates that this compound is hydrophobic (Vulava et al. 2016).

The fate of naproxen in the environment is affected by two phenomena: sorption and degradation (Liu et al. 2019; Martinez-Hernandez et al. 2016). The sorption process is strictly and inversely dependent on pH. Because naproxen has a carboxylic acid group that is deprotonated at an environmentally relevant pH (5–8), it occurs in the environment mainly in an anionic form. In this form, it can conjugate with the base forms in the aquatic and soil environments (Liu et al. 2019; Vulava et al. 2016). On the other hand, the electrostatic interactions of naproxen with negatively charged natural organic matter and clay mineral surfaces are difficult (Liu et al. 2019).

The basic changes that naproxen undergoes in surface waters are via its direct photochemical degradation and indirect photochemical pathways (Packer et al. 2003; Vulava et al. 2016). The intensity of photodegradation is affected by the intensity of light and the presence of nonorganic ions such as carbonate, nitrate, ferrous and ferric ions as well as organic matter, e.g. humic acids. Direct photochemical degradation of naproxen is possible because its UV absorption spectrum overlaps with the solar spectrum – > 290 nm (Sokół et al. 2017; Vulava et al. 2016). Its indirect photochemical degradation occurs when dissolved organic matter absorbs sunlight, which produces reactive oxygen species such as singlet oxygen, hydroxyl radicals or superoxide ions and other reactive species (Aguilar et al. 2019; Packer et al. 2003; Topp et al. 2008; Sokół et al. 2017; Vulava et al. 2016). Unfortunately, these processes lead to the formation of products that may be more persistent and more toxic (Vulava et al. 2016).

## The impact of naproxen on nontarget organisms

It is well known that pharmaceuticals present in the environment may have a negative ecotoxicological effect. Naproxen can affect the organisms inhabiting ecosystems either through its toxicity to an organism or via the toxicity of its metabolites. The latter can be formed during both physicochemical and biological processes (Jallouli et al. 2016; Rodriguez-Rodriguez et al. 2011). One of the most important aspects of studies on pollutant degradation is the increased toxicity of the degradation products. The photoderivatives of naproxen have been reported as being more toxic than the parent compound to *Brachionus calyciflorus*, *Thamnocephalus platyurus*, *Ceriodaphnia dubia*, *Vibrio fischeri* and *Daphnia magna *(DellaGreca et al. 2004; Jallouli et al. 2016). DellaGreca et al. (2004) showed that naproxen photoderivatives with a lower molecular weight such as the ethyl, carbinol, ketone and olefin derivatives are more active against bacteria than dimeric photoproducts. Moreover, the toxicity of its dimers is stereo-dependent (DellaGreca et al. 2004). Ma et al. (2014) reported that during simulated solar radiation, the generated product of naproxen photodegradation was more toxic than the parent compound. Its toxicity is probably connected with a loss of the chemical moieties of naproxen resulting in a lower steric effect and easier penetration into the cells of *Vibrio fischeri* (Ma et al. 2014). However, it was also demonstrated that the solar photocatalysis of naproxen partially reduces its acute toxicity (Jallouli et al. 2016; Marotta et al. 2013).

Many studies have indicated the negative effects of naproxen on aquatic invertebrates and vertebrates. It was shown that naproxen can accumulate in the bile of fishes where its concentration was 1000 times higher than this detected in samples of the lake (Brozinski et al. 2012). One explanation for the naproxen bioaccumulation may be the suppression of the metabolizing enzyme activity (Xu et al. 2019). Moreover, the presence of phase II metabolites in fish bile such as glucuronides was also detected. These intermediates undergo enzymatic deconjugation (Brozinski et al. 2012; Xu et al. 2019). It was also shown that naproxen at environmental concentrations may affect the mRNA expression and cause gastrointestinal and renal effects in zebrafish (Ding et al. 2017). A 14-day exposure to 10 μg/L of naproxen resulted in an altered gene expression in the gill tissue of zebrafish (Li et al. 2016). Li et al. (2016) observed that zebrafish embryos (LC_50_ = 115.2 mg/L) were more sensitive to naproxen than the larvae (LC_50_ = 147.6 mg/L). It was also shown that the larval zebrafish liver was particularly sensitive to naproxen. The hepatic reactions to the drug included a swelling of hepatic cells, hepatocellular vacuolar degeneration, and nuclei pycnosis and obscure cell borders were also observed. These reactions in fish may be connected with a modification of the organelles structure and an elevated stress level and could be a sign of the mobilization of an organism to detoxification. Moreover, a lower heart rate, pericardial oedema and teratogenic effects that are induced by naproxen may be connected with the inhibition of cyclooxygenases in *Danio rerio*. It is postulated that prostaglandins, which are products of the reactions that are catalysed by cyclooxygenases (COX), are necessary for proper heart formation (Li et al. 2016). It has also been postulated that naproxen may cause thyroid disruption in zebrafish because of the relatively high degree of similarity of the thyroid axis between humans and fishes. Xu et al. (2019) demonstrated a decrease in the thyroid hormone levels in zebrafish after exposure to naproxen. They postulated that this phenomenon resulted from a disturbance in the gene transcription along the hypothalamic-pituitary-thyroid axis and a significant decrease in transthyretin level (Xu et al. 2019).

It was shown a decrease in egg fertilization of *Jordanella floridae* over one complete life cycle occurred 121 days after exposure to 0.1 μg/L of naproxen. A low concentration of naproxen may also inhibit the growth of crustaceans such as *Ceriodaphnia dubia* after 7 days of exposure (Li et al. 2016).

Górny et al. (2019a) estimated the mean value of the microbial toxic concentration MTC_avg_, which is equivalent EC_50_ on the 1.66 g/L level using the MARA test with model organisms. This value indicated a low toxicity of naproxen for bacteria, which was probably connected with the lack of a proper carrier of naproxen in bacterial cells. Moreover, changes in the composition of the total fatty acids of *Bacillus thuringiensis* B1 were also observed (2015b). After incubation of B1 strain in the presence of naproxen, there was a significant increase in the value of the ratio of saturated and unsaturated fatty acids. The occurrence of the 16:0 iso 3OH fatty acid in bacterial cell membrane may stabilize its structure by interacting with the membrane protein (Górny et al. 2019a). The EC_50_ values that were estimated for *Chlorella vulgaris* and *Ankistrodesmus falcatus* were 40 mg/L after 24 h of exposure to naproxen (Ding et al. 2017). Ding et al. (2017) showed that the toxicity of naproxen on two algae, *Cymbella* species and *Scenedesmus quadricauda*, depended on its concentration and the duration of the incubation. The inhibition of growth increased together with the concentration of naproxen and decreased as the duration of the exposure increased. Moreover, a significant decrease in chlorophyll *a*, chlorophyll *b* and carotenoids was also observed. Naproxen decreased the number of enzymatic antioxidants, which led to a greater accumulation of OH^−^ and H_2_O_2_. At the same time, there was an increase of the malondialdehyde concentration. This compound may interact with biomolecules such as proteins, lipoproteins and DNA (Ding et al. 2017). Garcia-Medina et al. 2015 showed an increased superoxide dismutase (SOD) activity, which was connected with the generation of reactive oxygen species (ROS) after *Hyalella azteca* was exposed to naproxen. They suggested that the increase in SOD activity led to an increase in the hydrogen peroxide concentration and, as a result, to an increase in the activity of catalase. Oxidative stress damaged the genetic material of *Hyalella azteca* probably via the direct interaction of reactive oxygen species with DNA (Garcia-Medina et al. (2015). The genotoxicity of naproxen at a higher concentration was also observed by Górny et al. (2019a). However, this effect was not dose-dependent (Górny et al. 2019a).

Although low concentrations of naproxen occur in the environment, the increase in its toxicity may be related to a synergy effect with other contaminations. Zdarta et al. (2019) examined the toxicity of untreated and enzymatically treated solutions of naproxen and diclofenac against *Artemia salina*. However, they observed that after 24 h, the EC_30_ (the concentration of the drug at which 30% of the microorganisms showed a positive response after the exposure time) values for untreated naproxen and diclofenac solutions amounted around 20% and 25%, respectively. Enzymatic treatment with encapsulated laccase resulted in decreasing the EC_30_ values to around 80% and 85% for diclofenac and naproxen, respectively (Zdarta et al. 2019). A decrease in the toxicity was also observed by Marco-Urrea et al. (2010) and Rodriguez-Rodriguez et al. (2011) during the treatment of naproxen effluent using *Trametes versicolor*. A standard toxicity test using *Vibrio fischeri* showed a significant toxicity for naproxen at 10 mg/L only for the uninoculated control (EC_50_ amounted to 33% after 15 min of exposure) (Marco-Urrea et al. 2010).

Cleuvers (2004) showed that predicting the toxicity of a mixture is also indispensable because pharmaceuticals very rarely occur as a single contamination in the environment. When the concentrations of the non-steroidal anti-inflammatory drugs that are used in a mixture were compared to the individual compound, no observable effect concentrations (NOECs) of the single drugs revealed that considerable combination effect may occur even if substances were applied in concentrations below their NOEC (Cleuvers 2004). This observation was confirmed by Melvin et al. (2014) during research on *Limnodynastes peronei*. They observed an interactive effect of naproxen, carbamazepine and sulfamethoxazole on amphibian growth and development at environmental concentrations (Melvin et al. 2014). Jiang et al. (2017) showed that a mixture of naproxen, diclofenac and ibuprofen led to an increase in bacterial diversity in a sequencing batch reactor. According to the Shannon-Wiener diversity index, Actinobacteria and Bacteroidetes were enriched, whereas the number of *Micropruina* and *Nakamurella* decreased after the addition NSAIDs. Moreover, they observed damage in the cell wall of the microorganisms (Jiang et al. 2017). On the other hand, Grenni et al. (2014) showed that the chronic exposure to naproxen of the natural microbial community of the Tiber River caused a decrease in β-*Proteobacteria* especially ammonia-oxidizing bacteria and the *Archaea*. At the same time, an increase in α- and γ-*Proteobacteria* was observed. It is speculated that these last two are involved in naproxen biodegradation (Grenni et al. 2014). Due to the potential risk to organisms living in naproxen-contaminated environments, it is necessary to seek effective methods for its removal.

## Microbial decomposition of naproxen

**Fig. 2:**
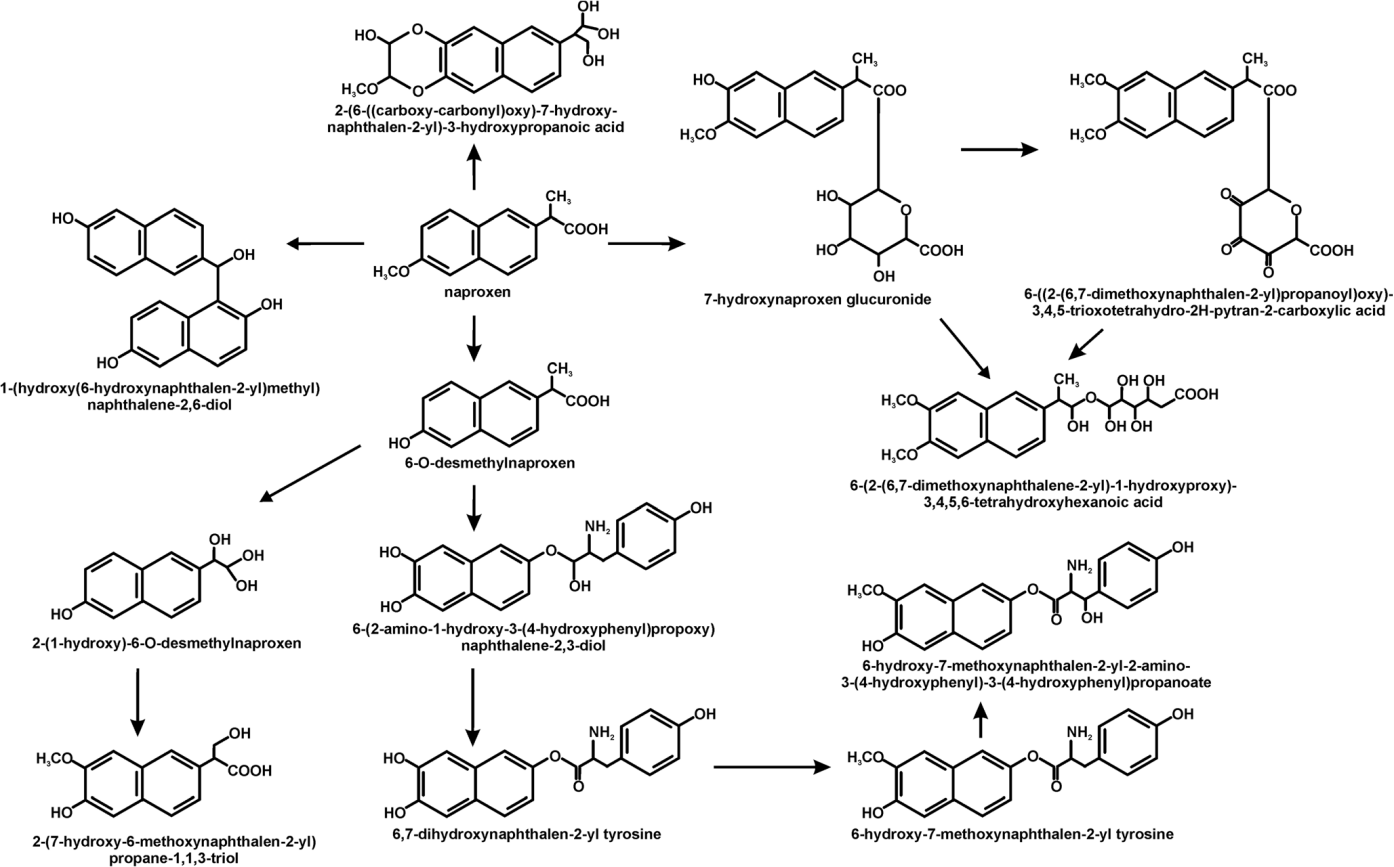
The decomposition pathways of naproxen in algae (Ding et al. 2017)

**Fig. 3:**
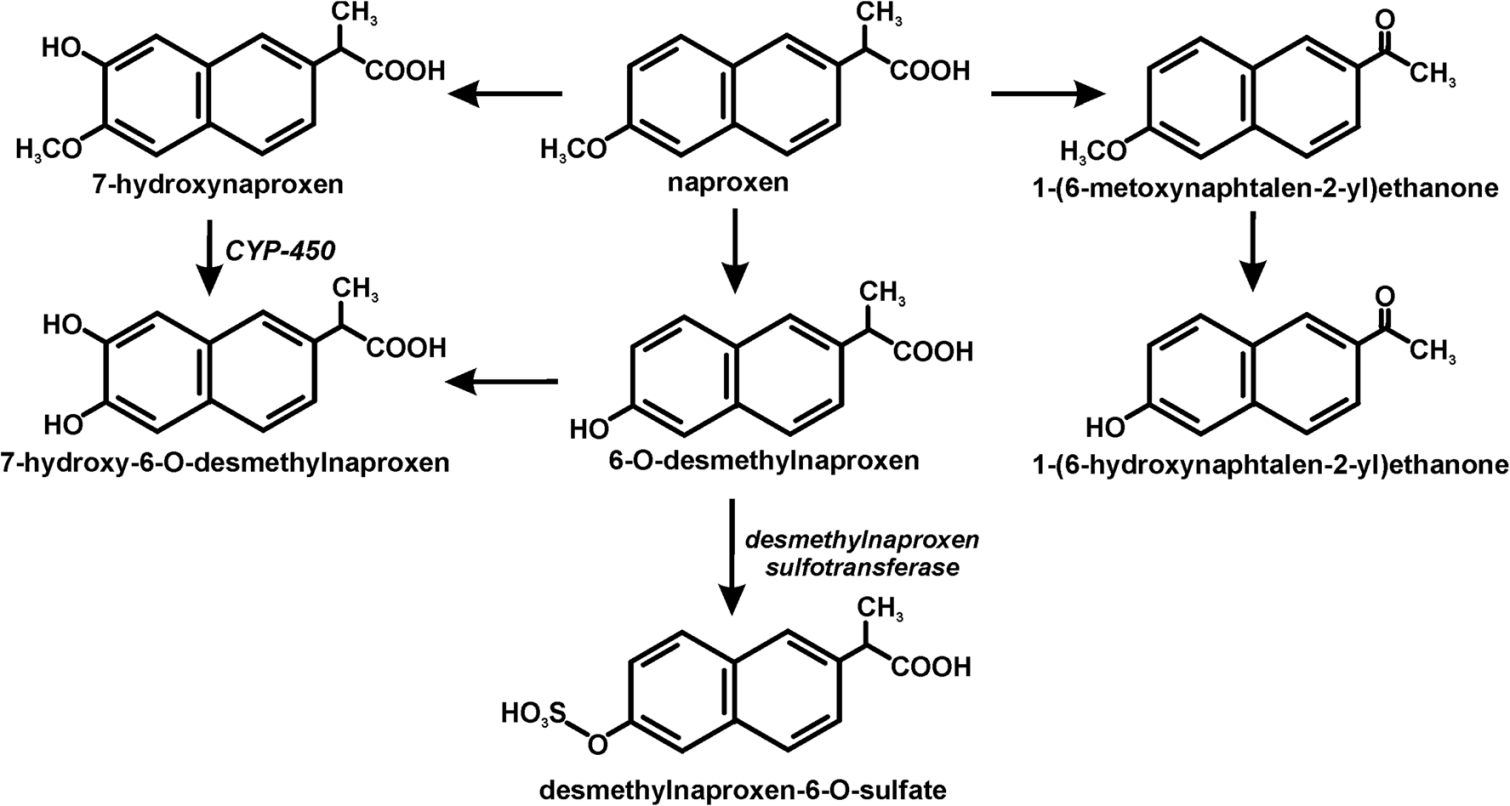
The decomposition pathways of naproxen in fungi (He and Rosazza 2003; Marco-Urrea et al. 2010; Qurie et al. 2013; Rodarte-Morales et al. 2012; Zhong et al. 2003)

Despite the increase in interest in the breakdown of non-steroidal anti-inflammatory drugs such as ibuprofen, ketoprofen and diclofenac in recent years, relatively little is known about the microbiological breakdown of naproxen. This is due to the relatively high stability of naproxen and its resistance to microbial degradation, which is connected with the presence of two condensed rings. This has been confirmed by the research that has been carried out to date. These show that most often there is only a microbiological transformation of naproxen, during which the aromatic rings are not cleaved (Domaradzka et al. 2015b). The transformation of naproxen is performed by bacteria, fungi and algae. Ding et al. (2017) observed the transformation of naproxen by the freshwater algae *Cymbella* sp. and *Scenedesmus quadricauda*. They identified 12 metabolites that had resulted from the hydroxylation, decarboxylation, demethylation, tyrosine conjugation and glucuronidation of naproxen (Fig. 2). Among the fungi, only *Aspergillus niger*, *Trametes versicolor*, *Phanerochaete chrysosporium*, *Myceliophthora thermophile*, *Cunninghamella blakeslesna* AS 3.153, *Cunninghamella echinulata* AS 3.2004, *Cunninghamella elegans* AS 3.156, *Bjerkandera adusta* and *Bjerkandera* sp. R1 can decompose naproxen (Jureczko and Przystaś 2018; Lloret et al. 2010; Marco-Urrea et al. 2010; Rodarte-Morales et al. 2011; Rodarte-Morales et al. 2012; Rodriguez-Rodriguez et al. 2010; Rodriguez-Rodriguez et al. 2011; Tran et al. 2010; Zhong et al. 2003). The transformation of naproxen by fungi is accompanied by the involvement of the extracellular oxidative enzymes such as laccase, manganese peroxidase, lignin peroxidase and versatile peroxidase (Lloret et al. 2010; Rodarte-Morales et al. 2012). Moreover, Marco-Urrea et al. (2010) showed that cytochrome P-450 might also play a role in the transformation of naproxen. The key metabolites that were observed included 7-hydroxynaproxen, 7-hydroxy-6-O-desmethylnaproxen, desmethylnaproxen, desmethylnaproxen-6-O-sulfate, 1-(6-hydroxynaphthalen-2-yl)ethanone, 1-(6-methoxynaphthalen-2-yl)ethanone (Fig. 3) (He and Rosazza 2003; Marco-Urrea et al. 2010; Qurie et al. 2013; Rodarte-Morales et al. 2012; Zhong et al. 2003). The efficiency of the transformation of naproxen by fungi is almost 100%. Moreover, Tran et al. (2010) showed that commercial laccase is more effective than crude laccase from *Trametes versicolor*.

**Fig. 4:**
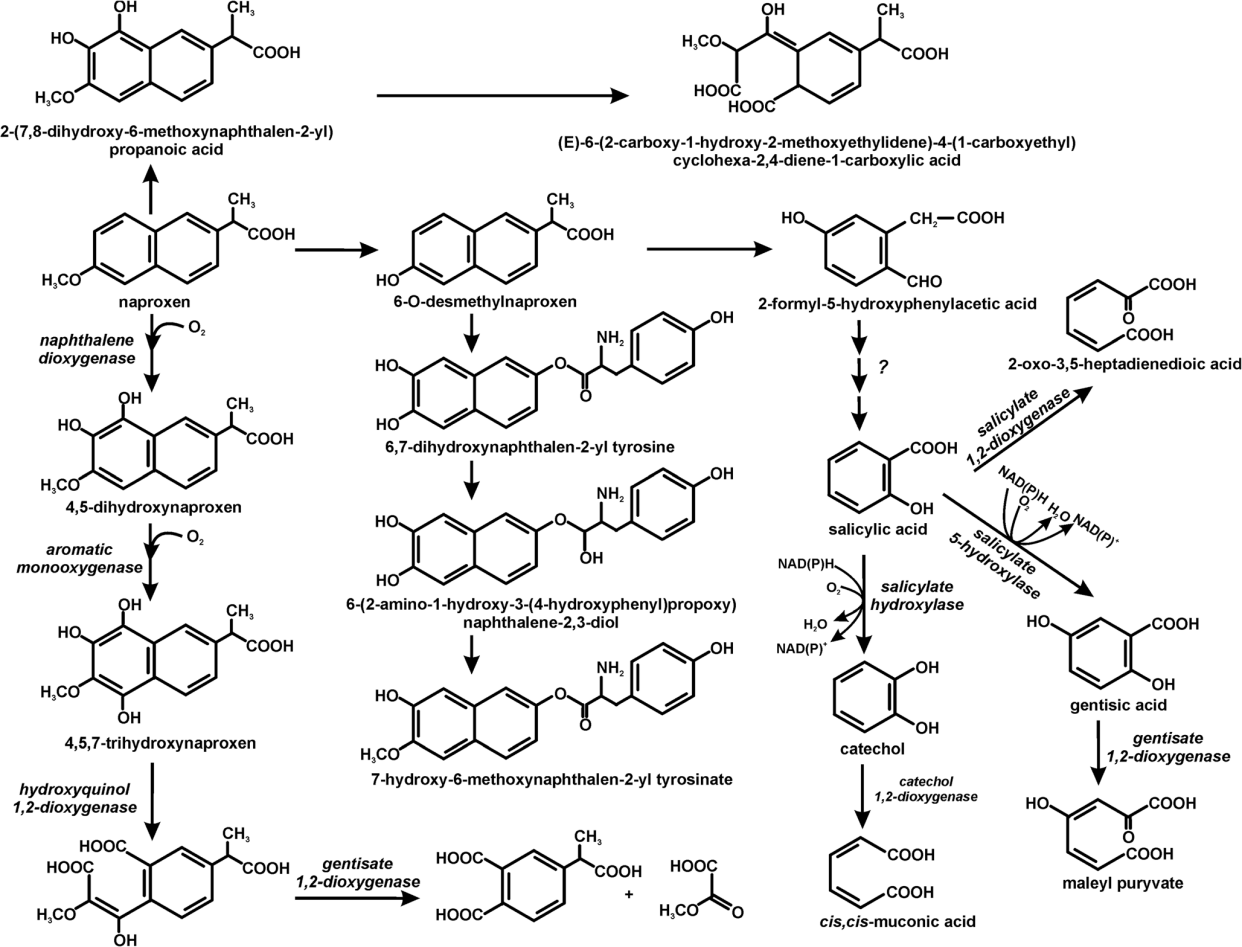
The biodegradation pathways of naproxen in bacteria (Domaradzka et al. 2015a; Górny et al. 2019b; Liu et al. 2019; Lu et al. 2019; Marchlewicz et al. 2016; Wojcieszyńska et al. 2014)

The total degradation of naproxen by pure strains was observed only in bacterial cultures: *Planococcus* sp. S5, *Bacillus thuringiensis* B1(2015b), *Stenotrophomonas maltophilia* KB2 and *Pseudoxanthomonas* sp. DIN-3 (Domaradzka et al. 2015a; Górny et al. 2019b; Lu et al. 2019; Marchlewicz et al. 2016; Wojcieszyńska et al. 2014). The degradation of naproxen in monosubstrate conditions occurred with a low efficiency. However, the presence of an additional carbon source such as glucose, phenol, 4-hydroxybenzoic acid or 3,4-dihydroxybenzoic acid increased the effectiveness of this process (Górny et al. 2019a; Wojcieszyńska et al. 2014). Similar results were obtained by Lu et al. (2019). In cometabolic conditions with acetate, glucose or methanol, the DIN-3 strain degraded naproxen with a higher efficiency than in monosubstrate conditions. Hydroxyquinol 1,2-dioxygenase is involved in the cleavage of naproxen in *Stenotrophomonas maltophilia* KB2 and *Planococcus* sp. S5 (Wojcieszyńska et al. 2014; Wojcieszyńska et al. 2016). Liu et al. (2019) proposed the transformation of naproxen by eliminating the methyl group. The O-desmethylnaproxen that is formed may conjugate with tyrosine to 6,7-dihydroxynaphthalene-2-yl-tyrosine. Naproxen can also be degraded via its hydroxylation to 2-(7,8-dihydroxy-6-methoxynaphthalene-2-yl) propanoic acid, which may subsequently undergo ring opening. The final product is (E)-6-(2-carboxy-1-hydroxy-2-methoxyethylidene)-4-(-1carboxyrthyl) cyclohexa-2,4-diene-1-carboxylic acid (Lu et al. 2019). The best described is the naproxen degradation pathway in *Bacillus thuringiensis* B1(2015b), which occurs via demethylation to O-desmethylnaproxen. This metabolite is converted to 2-formyl-5-hydroxyphenyl-acetate. Another identified metabolite of this pathway is salicylic acid, which is cleaved by salicylate 1,2-dioxygenase or is hydroxylated to catechol or gentisic acid. The pathway with catechol as an intermediate, which is cleaved to *cis,cis*-muconic acid involved in central metabolism, probably dominates in this strain (Górny et al. 2019b). The microbial pathways of naproxen decomposition are presented in Fig. 4.

Studies with mixed populations of microorganisms indicate a higher efficiency of naproxen degradation using monocultural systems. The natural microbial community in the Tiber River degraded 0.1 mg/l naproxen within about 40 days, although there was a transient negative effect on this community (Grenni et al. 2014). Moreover, Caracciolo et al. (2012) observed the ability of an autochthonous bacterial community in river water to completely eliminate naproxen over the course of 30 days. In turn, Quintana et al. (2005) showed that in the presence of powdered milk, 20 mg/l of naproxen was transformed by 50% by the active sludge in a membrane bioreactor. It was also demonstrated that naproxen degradation can occur under anaerobic conditions (Lahti and Oikari 2011).

## Conclusion

Analysis of the current state of knowledge indicates that due to the increasing intake of naproxen, the problem of its occurrence in the environment will continue to increase. Toxicological studies indicate that long-term exposure to environmental doses may negatively affect the organisms that live in a habitat, especially if naproxen co-occurs with other drugs. The vast majority of literature reports indicate the transformation of naproxen without decomposition of condensed aromatic rings. Another problem is the appearance of hydroxylated derivatives of naproxen as a result of transformation, which may have a more negative impact on living organisms, due to their greater hydrophilicity. To date, only a few bacterial strains possessing enzymes of the full naproxen degradation pathway have been described. However, compared to monocyclic non-steroidal anti-inflammatory drugs such as ibuprofen, the level of naproxen degradation is definitely lower. Therefore, it is still necessary to search for the strains and consortia of microorganisms that have an increased potential to degrade naproxen.
